# Genome‐wide association study for Alzheimer's disease related complement components and regulators

**DOI:** 10.1002/alz70855_107268

**Published:** 2025-12-25

**Authors:** Samantha Clayton, Gyungah R Jun

**Affiliations:** ^1^ Boston University School of Medicine, Boston, MA, USA; ^2^ Department of Medicine (Biomedical Genetics), Boston University Chobanian & Avedisian School of Medicine, Boston, MA, USA

## Abstract

**Background:**

Alzheimer's disease (AD) is a progressive neurodegenerative disorder characterized by buildup of amyloid‐beta plaques and tau tangles. Previous studies demonstrate involvement of complement pathway in AD. Investigating genetic factors influencing plasma levels of complement pathway genes may uncover critical mechanisms underlying AD.

**Method:**

We first conducted association of plasma levels of complement related proteins with domain specific cognitive scores including executive function, language, and memory scores in 2,476 Framingham Heart Study (FHS) participants. We conducted genome‐wide association studies (GWAS) with the selected complement proteins as quantitative outcomes, adjusting for age at exam, sex, family structure, and three principal components included as covariates. We excluded loci around genes encoding testing complement proteins.

**Result:**

We observed the 29 proteins with nominally significant associations (*p* <0.05) with at least one domain‐specific cognitive scores (best P with C1r and executive function: *p* = 1.45x10^‐11^). VTN, MBL2, FCN3, CFI, C8, C5, and C1r were nominally significant with both executive function and language. C5 and MBL2 were negatively associated with these scores, while the other five had increased expression with higher scores. We conducted 29 GWASs and identified 57 genome‐wide significant (GWS) single nucleotide polymorphisms (SNPs). Intronic SNP, rs147931340, from *GPSM1* was GWS across different complement proteins including C1r (*p* = 6.12x10^‐13^), C8 (*p* = 5.00x10^‐10^), FCN3 (*p* = 2.05x10^‐11^), and MBL2 (*p* = 4.36x10^‐12^). An intergenic SNP rs28378835 between ONECUT3 and TCF3 was GWS for C1r (*p* = 5.87x10^‐12^), FCN3 (*p* = 6.65x10^‐9^), and MBL2 (*p* = 1.17x10^‐8^). These variants were associated with increased expression with all but MBL2.

**Conclusion:**

Our study identified significant associations between plasma levels of complement proteins and domain‐specific cognitive scores, highlighting their potential relevance to Alzheimer's disease (AD) pathology. These findings provide valuable insights into the genetic regulation of complement pathways in AD and suggest potential mechanisms underlying their role in cognitive decline.

